# Age- and sex-related differences in human α4β2* nicotinic acetylcholine receptor binding evaluated with [ ^18^ F]nifene PET

**DOI:** 10.1162/imag_a_00397

**Published:** 2024-12-16

**Authors:** Andrew McVea, Jihoon Choi, Alexandra DiFilippo, Max McLachlan, Brecca Bettcher, Matthew Zammit, Charles K. Stone, Dana Tudorascu, Jogeshwar Mukherjee, Bradley T. Christian

**Affiliations:** Department of Medical Physics, University of Wisconsin — Madison School of Public Health, Madison, WI, United States; Waisman Laboratory for Brain Imaging and Behavior, University of Wisconsin — Madison School of Public Health, Madison, WI, United States; Wisconsin Alzheimer’s Disease Research Center, University of Wisconsin — Madison School of Medicine and Public Health, Madison, WI, United States; Department of Psychiatry, University of Pittsburgh, Pittsburgh, PA, United States; Department of Radiological Sciences, University of California — Irvine School of Medicine, Orange, CA, United States

**Keywords:** PET imaging, thalamic nuclei, distribution volume ratio (DVR), nAChR imaging, reference region

## Abstract

Neuronal α4β2* nicotinic acetylcholine receptors (nAChRs) are stimulated by nicotine and are associated with tobacco dependence. [^18^F]Nifene is a PET radiotracer with high specificity for α4β2* nAChRs that can be used to investigate nAChR distribution in the human brain*in vivo*. In this study, we investigate the dependence of sex and age on the binding of [^18^F]nifene in nonsmoking healthy human participants. Cognitively normal participants (n = 31) were recruited into older versus younger and male versus female cohorts to investigate sex and age differences in [^18^F]nifene binding. Distribution volume ratios (DVRs) were calculated for brain regions with known nAChR expression and compared using a multiparameter linear regression model. There was a significant association between age and decreasing thalamic DVR (p = 0.01), with the most notable difference coming from the anterior nucleus of the thalamus (p < 0.001). Outside of the thalamus, a higher [^18^F]nifene DVR was observed with increasing age in the cerebellar grey matter (p = 0.01). No significant sex differences were observed using our linear model after multi-comparison correction. These results support including age in the experimental design and analysis of the α4β2* nAChR system in research and clinical applications.

## 1 Introduction

Nicotinic acetylcholine receptors (nAChRs) are ligand-gated ion channels that respond to nicotine and acetylcholine. In the brain, they function as excitatory neurotransmitter receptors that allow a flux of cations when activated and correspondingly impact synaptic activity by influencing the release of neurotransmitters (McKay et al., 2007). While nAChRs can take various subunit configurations, the most common combinations are known as α4β2*, which includes the β2 subtypes α4β2, α3β2, and α2β2 (Lindstrom et al., 1995). These receptors have been implicated in a variety of neurodegenerative and neuropsychiatric disorders, including Alzheimer’s disease (AD), Parkinson’s disease (PD), and nicotine addiction.

Sex differences have been observed in the incident rates of AD and other types of dementia, especially in older populations with higher disease rates seen in women (Beam et al., 2018). Similarly, studies have demonstrated that women, especially in younger cohorts, are more susceptible to nicotine addiction and have worse cessation outcomes (O’Dell & Torres, 2014). Compositional, hormonal, and metabolic sex and age differences that affect the mechanistic regulation of nAChRs have been proposed to explain these differences (Benowitz & Hatsukami, 1998;Pogun et al., 2017), including changes in estrogen and progesterone, which can modulate nAChRs and may contribute to the sex and age differences observed in nicotine addiction (Cosgrove et al., 2007). Variations in behavioral and clinical outcomes across phases of the estrous cycle in animal models and menstrual cycle in humans indicate a potential mediating role of female sex hormones in nAChRs regulation (Cosgrove et al., 2012), further emphasizing the importance of considering sex and age in nAChR studies.

Various laboratory techniques, such as ligand binding assays, autoradiography, and immunoblot, have been used to study nicotinic receptors. With the development of neuroimaging methods enabling*in vivo*visualization and dynamic monitoring of nAChRs, single-photon emission computed tomography (SPECT) and positron emission tomography (PET) have been integrated into exploring their physiology within the brain. Human PET studies have demonstrated alterations in the binding potential of nAChR radiotracers in the brains of individuals with neurodegenerative disorders and cognitive decline (Meyer et al., 2014). In people with AD, a reduction in binding was associated with a decline in executive function and with aging (Colloby et al., 2010;Okada et al., 2013). A similar pattern was observed in PD without dementia and dementia with Lewy bodies (Meyer et al., 2009;O’Brien et al., 2008). These findings implicate a potential role of nAChRs for modulating synaptic regulation in neurodegenerative disorders (McKay et al., 2007).

[^18^F]Nifene is a PET radioligand with high specificity for a4β2* nAChRs and has been shown to correlate to the*in vivo*density of nAChRs expression in preclinical models (Hillmer et al., 2012;Pichika et al., 2006), providing information on the distribution, availability, and activity of nAChRs in the brain (Mukherjee et al., 2018). [^18^F]Nifene demonstrates faster*in vivo*kinetics, reaching (pseudo) equilibrium within 40 min (Lao et al., 2017), compared to other nAChR targeting agents that require 1.5 h to over 6 h for accurate quantitation as observed in 2-[^18^F]A85380 (2-FA), [^18^F]AZAN, [^18^F]flubatine, and [^18^F]XTRA (Chefer et al., 2003;Cosgrove et al., 2007;Coughlin et al., 2018;Fujita et al., 2002,^2003^;Horti et al., 1998,^2010^,^2013^;Kimes et al., 2003;Sabri et al., 2015;Wong et al., 2013). It is speculated that the longer equilibrium times of these radioligands are the result of a slow trapping component of these ligands within intracellular acidic organelles, which has been shown both in vitro and in vivo studies (Zammit et al., 2023;Zhang et al., 2023). The moderate binding affinity for nAChRs and favorable imaging kinetics of [^18^F]nifene offer an advantage for research investigations requiring comparatively short acquisition times in brain regions with high receptor density.

Previous first-in-human studies using [^18^F]nifene PET demonstrated that it was well tolerated by all participants with no adverse events reported, and revealed a test-retest variability within 10% (Betthauser et al., 2017;Lao et al., 2017), which is comparable to most PET neuroligands. These studies were conducted on a cohort of eight healthy participants with an equal proportion of male and female individuals; however, they were not sufficiently powered to thoroughly examine age and sex-based differences. Investigations with [^18^F]2-FA and [^18^F]XTRA demonstrated an inverse correlation between age and nAChR binding, with no significant sex-differences reported in the [^18^F]XTRA study (Coughlin et al., 2018;Sultzer et al., 2022). The goal of this investigation was to examine sex- and age-dependence on the α4β2* nAChR binding of [^18^F]nifene using a larger sample size and wider age range to provide further insight into demographic trends for the expanded use of [^18^F]nifene for human clinical research investigations.

## 2 Methods

### 2.1 Study participants

Human PET/CT and MRI studies were carried out on healthy volunteers under an FDA approved Investigational New Drug (IND) application and approved by the Institutional Review Board (IRB) of University of Wisconsin-Madison. This study was designed to enroll healthy, nonsmoking male and female participants into younger (age <30 years) and older (age >60 years) cohorts. Potential participants were recruited from the University of Wisconsin - Madison research database, and the study was also advertised to the public at the University of Wisconsin campus and through emails sent to students and faculty. Exclusion criteria for participants included a history of psychiatric or neurodegenerative diseases, current pregnancy or lactation, previous hormone replacement therapy, history of nicotine use, and use of prescription psychotropic drugs. Nicotine use status was verbally reported by participants prior to enrollment in the study. Written consent was obtained prior to any participant procedures according to the Declaration of Helsinki.

A total of 31 participants across 4 cohorts (Table 1) underwent [^18^F]nifene and MRI scans. Included in this group were six participants from a previous human study conducted with [^18^F]nifene at our site that fell within these specified age ranges.

**Table 1.** Description of [^18^F]nifene imaging cohort.

Cohort	Participants (n)	Previous study (n)	Age (years)	Age range (years)
Younger men	7	1	24.6 ± 2.9	20-28
Younger women	6	2	22.6 ± 1.7	20-25
Older men	6	1	66.7 ± 5.1	60-77
Older women	6	2	69.5 ± 4.5	59-74
Total	25	6	45.2 ± 22.6	20-77

One younger male participant was excluded from group analysis due to recreational use of nicotine pouches prior to imaging discovered after completion of the imaging protocol. An identical imaging and processing procedure was completed for this participant and is included with the results labeled as “nicotine user”.

### 2.2 MRI scans

T1 weighted MRI were acquired for all participants for anatomical reference and image registration. They were acquired on a 3.0T SIGNA 750 (GE) using a spoiled gradient sequence (TI/TE/TR:450/3.2/8.2 ms, flip angle:12°, slice thickness:1 mm no gap, FOV:256, matrix dimensions: 256 × 256 × 156).

### 2.3 PET imaging

[^18^F]Nifene used in this study was synthesized at our site using previously published methods from our group (Hillmer et al., 2011). All participants were injected with an average of 6.8 ± 0.8 mCi of [^18^F]nifene with a corresponding average injected mass of 0.24 ± 0.14 μg and imaged from 0–60 min at our site at the University of Wisconsin. The study participants were imaged on a Biograph Horizon mCT (4 ring), while the six participants from our previous study were imaged on a Siemens ECAT EXACT HR+. PET images consisting of 21 frames (8 x 30 s, 3 x 120 s, 10 x 300 s) were reconstructed using filtered back projection with a ramp filter (image size:128 × 128 × 63, voxel size: 2.57 × 2.57 × 2.43 mm) for images on the ECAT HR+ and using iterative reconstruction (4i,8sub) with an all-pass filter (image size: 360 × 360 × 109, voxel size: 1.03 × 1.03 × 2.03 mm) on the Biograph PET/CT.

### 2.4 Data analysis

T1 weighted MRI were skull stripped using FSL (Woolrich et al., 2009) and used as anatomical reference to bring PET images into standard space. In native space, the individual PET frames were corrected for interframe motion during the course of the scan using SPM12 (Ashburner et al., 2011), and denoised by smoothing temporally using the HYPR-LR algorithm (Christian et al., 2010). Images were then converted into Distribution Volume Ratio (DVR) images using the Logan reference tissue method (Logan et al., 1996) at pseudo-equilibrium without a$\bar{k2}$term using a t* = 10 min and an eroded corpus callosum (CC) ROI as a reference region. Logan reference tissue modeling was chosen for this study due to its wide use in DVR calculation without blood sampling and the low noise profile of the data after denoising. Previous work in our group (Hillmer et al., 2012) found close agreement between Logan and other DVR estimation methods, including the multilinear reference tissue model (MRTM). The DVR images were then registered to the participant MRI using a rigid body transform and then into standard space using an affine transform calculated from the spatially normalized skull stripped MRI. Using the PET scanner harmonization methods described inLodge et al. (2018), the DVR images in standard space were then smoothed using a 3D gaussian smoothing kernel (5.85 mm^3^for Biograph PET/CT, 5.44 mm^3^for ECAT HR+) to achieve uniform image smoothness for group analysis.

Previous work with nAChR PET tracers validated the CC as a reference region (Brody et al., 2006;Hillmer et al., 2013) due to the lack of specific binding in this brain area and the widespread uptake of the tracer elsewhere throughout the cortices. Using the CC as a reference region can be challenging for age-based comparisons due to its small volume and the proximity of this region to the ventricles. Ventricle enlargement is typically seen with normal aging (Kaye et al., 1992), and as this brain region with low PET signal expands it can begin to encroach into the CC ROI and decrease the reference region signal. Initially, our reference region was defined using the Morimod WM atlas (Oishi et al., 2008) smoothed by 6 mm isotopically and eroded to only include voxels with a value >0.95. Using this method, we observed a large overlap between the CC ROI and the ventricles in 10 older participants (67% of the total), significantly decreasing the reference region average signal for these participants and leading to a higher global DVR.

**Fig. 1.**
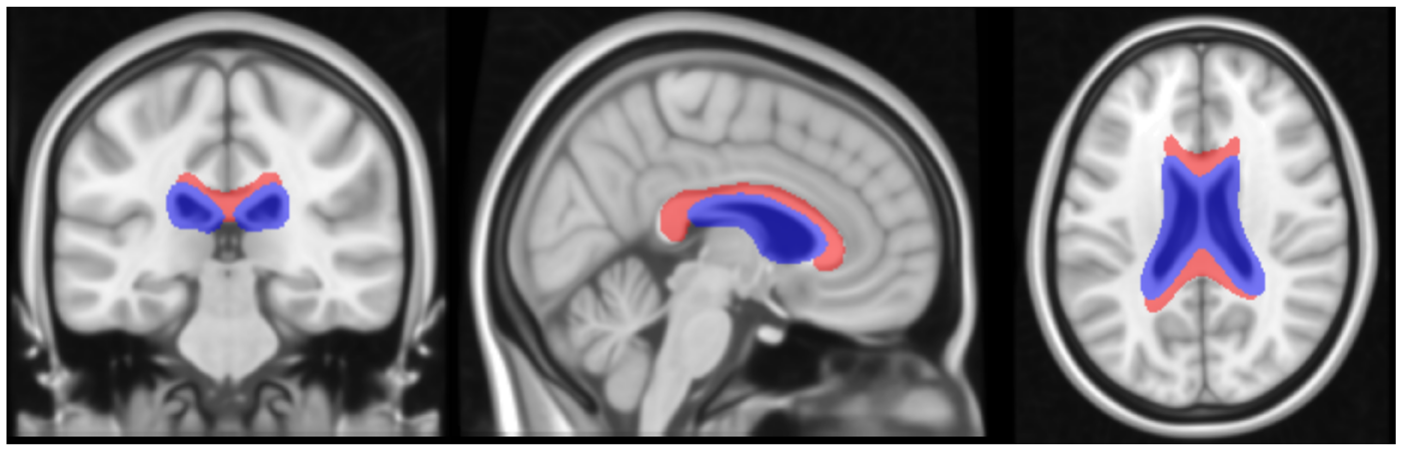
Brain slices visualizing the CC reference region used in this analysis. The final reference region is shown in red, the ventricle mask in blue.

In order to account for the overlap between the CC ROI and enlarged ventricles seen in our older participants, a uniform volume CC reference region was created based on the Morimod atlas with areas of common ventricle overlap removed in standard space. To accomplish this, the ventricles for each participant were first segmented using Freesurfer (v7.1.1) in native space. An affine transform was applied to bring the participant MRIs and individual ventricle segmentations into standard space. The individual ventricle segmentations were then averaged together such that a voxel included in all of the segmentations was assigned a value of 1, a voxel included in 50% of the segmentations was given a value of 0.5, and a voxel with no overlap had a value of 0. All voxels in standard space with a weight greater than 0.25, indicating an overlap of the ventricles in 25% of participants, were then included in our ventricle mask. The ventricle mask was then subtracted from the Morimod CC mask to create the final reference region (Fig. 1), which has a uniform volume of 22.4 cc in standard space for each participant. This common space CC reference region was spatially transformed into MRI space using the inverse affine transform and then into native PET space using an inverse rigid body transform calculated from the coregistration of the PET and MRI images for each participant in order to calculate the DVR parametric image.

The analysis focused on the regions of the brain with the highest a4β2* nAChR expression as well as large cortical and subcortical areas with moderate receptor expression. Four subregions of the thalamus with the greatest receptor expression were defined using the human thalamic nuclei brain atlas (Saranathan et al., 2021): the anterior nucleus (AN), lateral nucleus (LN), medial nucleus (MN), and posterior nucleus (PN), which includes the lateral geniculate nucleus (LGN), medial geniculate nucleus (MGN), and pulvinar. Extrathalamic regions for analyses were identified using the Harvard-Oxford Atlas (Desikan et al., 2006;Frazier et al., 2005;Goldstein et al., 2007;Makris et al., 2006) consisting of the striatum, cerebellar grey matter, hippocampus, and frontal lobe. Additionally, a fixed-size ROI drawn in standard space covering the ventral tegmental area and substantia nigra (SN) previously described byLao et al. (2017)was included in analysis as one region due to their small sizes with a combined volume of 2.02 cc.

Comparisons of regional [^18^F]nifene between age and sex cohorts were completed using a multiparameter linear regression model in R incorporating sex, age, and the interaction term between sex and age to calculate p-values. Although our regions were selected a-priori, False Discovery Rate (FDR) multi-comparison correction was applied to these p-values to determine the α level for significance for each comparison. The linear model has the functional form$DVR=\beta_{0}+\beta_{1}*Age+\beta_{2}*Sex+\beta_{3}*Age*Sex$. The β values are calculated for the linear model using R and give the intercept of the linear fit (β_0_), contribution from age (β_1_), sex (β_2_), and the interaction between sex and age (β_3_). Note that for this modelSex=0for men andSex=1for women.

## 3 Results

The highest [^18^F]nifene binding was observed in the brain regions with the known highest a4β2* nAChR density in the thalamus (Table 2), most notably in the MN, as well as in the SN across all age and sex cohorts.

**Table 2.** Group comparisons in [^18^F]nifene binding (DVR).

Region	Total	Nic (n = 1) *	Older	Younger	Male	Female	β _0_ [CI]	β _1_ [CI]	β _2_ [CI]	β _3_ [CI]	Age (p)	Sex (p)
Thalamus	2.23 ± 0.30	1.37	2.06 ± 0.24	2.39 ± 0.26	2.28 ± 0.28	2.18 ± 0.31	2.53 [2.23, 2.82]	-0.008 [-0.013, -0.002]	0.06 [-0.38, 0.50]	0.001 [-0.008, 0.009]	**0.01**	0.78
Striatum	1.47 ± 0.12	1.41	1.48 ± 0.14	1.46 ± 0.11	1.48 ± 0.12	1.47 ± 0.13	1.49 [1.34, 1.63]	-0.000 [-0.003, 0.002]	-0.08 [-0.30, 0.13]	0.002 [-0.002, 0.006]	0.76	0.43
Cer GM	1.47 ± 0.18	1.35	1.54 ± 0.19	1.40 ± 0.13	1.46 ± 0.14	1.48 ± 0.21	1.24 [1.05, 1.42]	0.005 [0.002, 0.009]	0.15 [-0.12, 0.43]	-0.003 [-0.009, 0.002]	**0.01**	0.26
Hipp	1.44 ± 0.14	1.36	1.48 ± 0.16	1.39 ± 0.11	1.44 ± 0.12	1.43 ± 0.16	1.27 [1.12, 1.43]	0.003 [0.000, 0.006]	0.12 [-0.12, 0.35]	-0.002 [-0.007, 0.002]	0.03	0.32
Frontal	1.36 ± 0.15	1.37	1.40 ± 0.17	1.33 ± 0.12	1.36 ± 0.13	1.36 ± 0.16	1.27 [1.10, 1.45]	0.002 [-0.001, 0.005]	0.03 [-0.23, 0.28]	-0.001 [-0.006, 0.005]	0.23	0.84
Substantia nigra/ventral tegmental area	1.63 ± 0.22	1.20	1.62 ± 0.22	1.64 ± 0.22	1.64 ± 0.21	1.62 ± 0.23	1.53 [1.27, 1.79]	0.002 [-0.003, 0.007]	0.22 [-0.16, 0.60]	-0.004 [-0.012, 0.003]	0.41	0.25
Anterior nucleus	1.82 ± 0.60	1.32	1.52 ± 0.51	2.10 ± 0.55	1.80 ± 0.45	1.83 ± 0.72	2.89 [2.33, 3.44]	-0.023 [-0.034, -0.012]	-1.03 [-1.85, -0.21]	0.022 [0.005, 0.038]	**< 0.001**	0.02
Lateral nucleus	2.31 ± 0.30	1.32	2.32 ± 0.30	2.30 ± 0.31	2.30 ± 0.29	2.31 ± 0.31	2.21 [1.85, 2.57]	0.002 [-0.005, 0.009]	0.16 [-0.37, 0.69]	-0.004 [-0.014, 0.007]	0.50	0.55
Posterior nucleus	2.35 ± 0.39	1.44	2.19 ± 0.31	2.50 ± 0.40	2.47 ± 0.38	2.24 ± 0.37	2.50 [2.09, 2.91]	-0.006 [-0.014, 0.002]	0.30 [-0.30, 0.91]	-0.002 [-0.014, 0.010]	0.15	0.32
Medial nucleus	2.71 ± 0.44	1.48	2.48 ± 0.34	2.93 ± 0.41	2.78 ± 0.45	2.65 ± 0.41	3.02 [2.57, 3.46]	-0.008 [-0.016, 0.001]	0.30 [-0.35, 0.96]	-0.004 [-0.017, 0.009]	0.06	0.35

* Participant using recreational nicotine. Significant p-values are indicated in bold.

### 3.1 Age-related differences

**Fig. 2.**
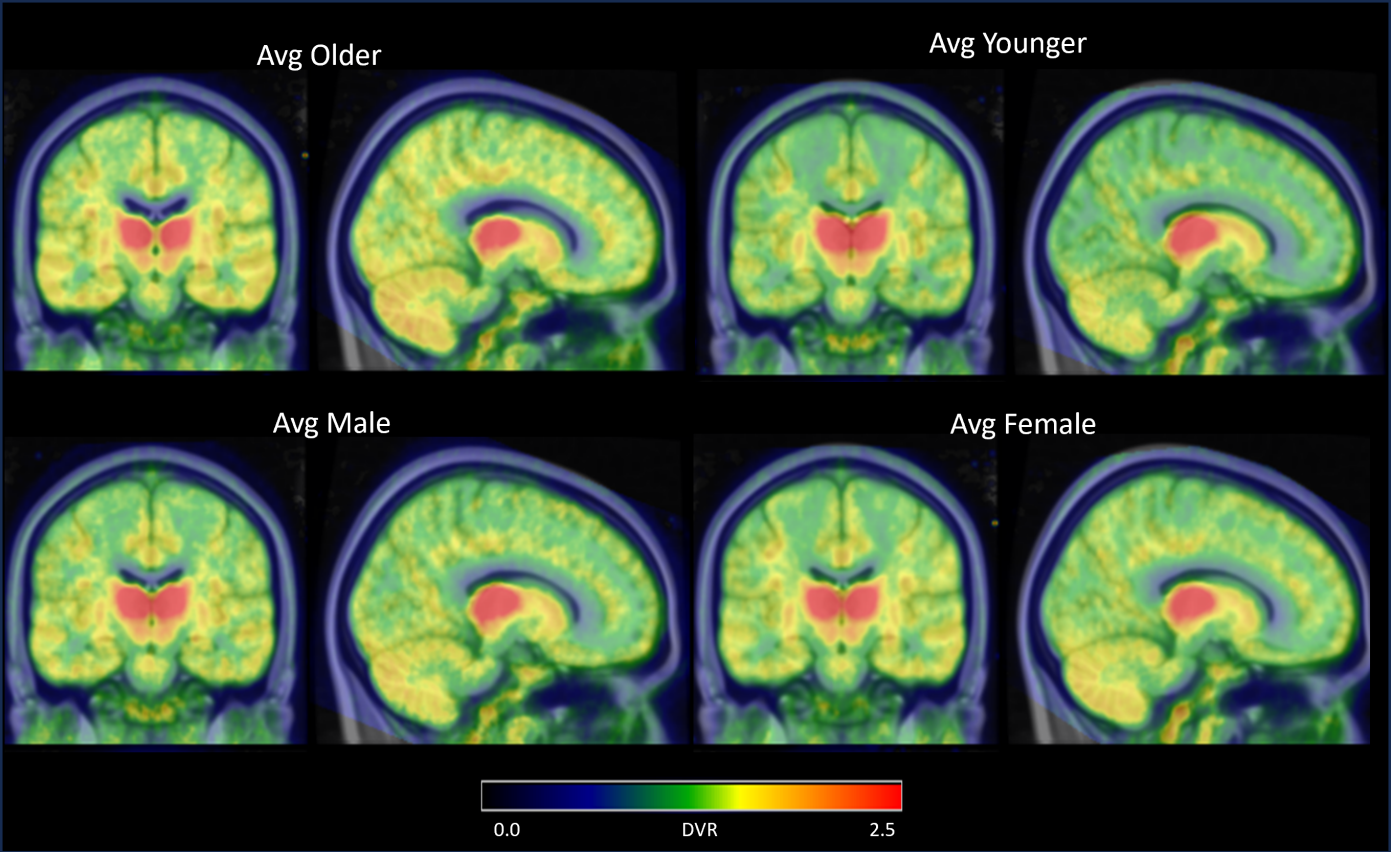
Group average for the older (top left), younger (top right), male (bottom left), and female (bottom right) cohorts analyzed in this study.

**Fig. 3.**
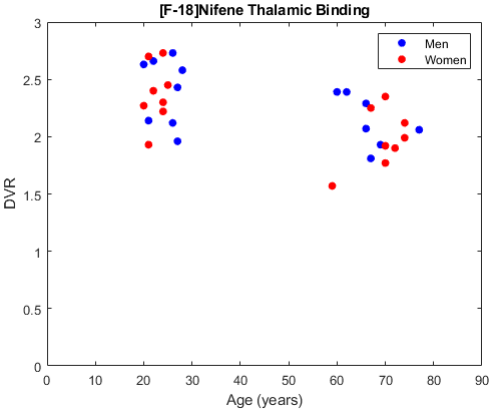
Scatter plot showing the difference in full thalamus DVR against age in our imaging cohort. A clear decrease in [^18^F]nifene binding is observed on average between the younger and older cohorts with increasing age.

Comparing the younger and older cohorts (Fig. 2), a higher average DVR is found in the full thalamus of the younger cohort (2.38 ± 0.26) compared to the older cohort (2.05 ± 0.26) (Fig. 3). Partitioning the thalamus into its component nuclei, higher [^18^F]nifene binding is observed in the younger cohort throughout most of the thalamic nuclei, including the AN, MN, and PN (Table 2). Using FDR multicomparison correction on the p-values an α for signficance for age-related effects was set to 0.02. Using the statistical model to compare the age-associated changes in DVR, a significant interaction between age and thalamic DVR (p = 0.01) is observed with DVR decreasing with age. This difference is primarily driven by the AN (p < 0.001), which has a DVR of 1.52 ± 0.51 and 2.10 ± 0.55 for the older and younger cohorts respectively.

Outside of the thalamus, we observe a significant age-related association with the cerebellar grey matter DVR (p = 0.01) with an increase observed with higher age. The older cohort reported an average DVR of 1.54 ± 0.19 compared to 1.40 ± 0.13 in the younger cohort in the cerebellar grey matter.

### 3.2 Sex differences

While the male and female populations in our study had similar mean DVR values in the AN of the thalamus, women on average had a higher value (1.83 ± 0.72) compared to men (1.80 ± 0.45) in our statistical model (p = 0.02). While the thalamus and its component nuclei were chosen a priori, the p-values for sex-related differences in any of the regions analyzed were not significant after FDR multi-comparison correction with an α = 0.005. The AN of the thalamus also revealed age-dependent differences in [^18^F]nifene binding with an interaction term between age and sex (p = 0.01), but similarly did not reach the threshold for significance. The linear regression derived for this region was$DVR_{AN}=2.89-0.02*Age-1.03*Sex+0.02*$Age*Sexwith a standard error of 0.27, 0.01/year, 0.40 and 0.01/year for the intercept, age, sex, and interaction components respectively. There were no significant differences in any regions analyzed with neither group displaying consistently higher binding across brain regions.

### 3.3 Nicotine user comparison

**Fig. 4.**
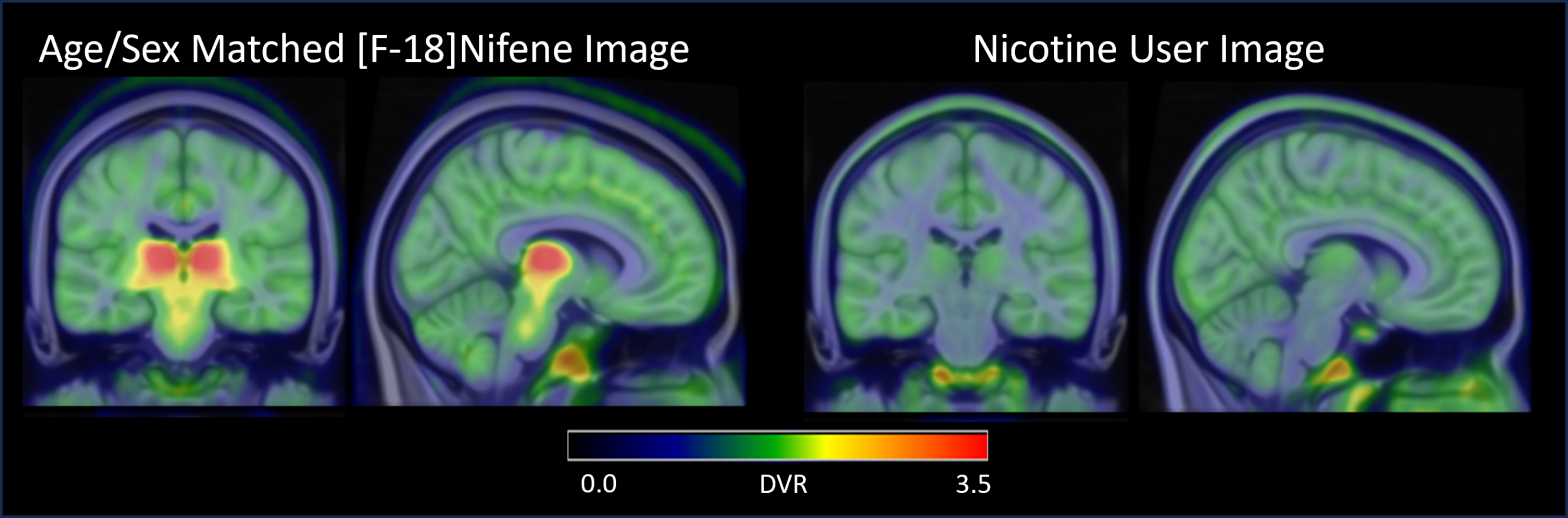
[^18^F]nifene images for two male 26 year-old participants. One had a typical [^18^F]nifene distribution (left) with highest binding in the thalamus, and one used recreational nicotine products prior to imaging (right) and shows primarily nonspecific binding.

Comparing the nicotine user participant to an age- and sex-matched participant in this study (Fig. 4), we observe competitive binding between the nicotine in their system and the [F-18]nifene. Visually, there is a clearer delineation between white matter and grey matter in the nicotine user participant than what is seen in the study cohort and a notable reduction in signal in regions known for high nAChR density, including the thalamus and SN. Comparing the DVR of the nicotine user participant to the average of our imaging population (Table 2), the DVR of the nicotine user participant are within one standard deviation of the population mean in all regions except for the thalamus, thalamic nuclei, and SN. The standardized uptake value (SUV) from 40–60 min in the CC of the nicotine user (1.37) is reduced compared to the other younger male participants (2.27 ± 0.12) included in this study. This is a difference of over 7 standard deviations, and the nicotine user has an SUV outside the 95% confidence interval [2.17,2.37] of the younger male distribution.

## 4 Discussion

Nicotine abuse through smoking is the leading preventable cause of disease and death in the United States (U.S. Department of Health and Human Services, 2014), and differences in the mechanisms of nicotine addiction and cessation between men and women have been previously observed (Verplaetse et al., 2018). The density and stimulation of nAChRs have also been found to have associations with cognition and memory (Terry & Callahan, 2019), and a decrease in these receptors was demonstrated in individuals with cognitive decline (Meyer et al., 2014). The regions of highest [F-18]nifene binding were observed in the thalamus, specifically the MN, which had an average DVR of 2.71 ± 0.44 across our imaging cohort. This finding replicates previous studies examining nAChR binding using PET imaging that also found the highest density of nicotinic receptors in the thalamic regions projecting to the prefrontal cortex, including the mediodorsal nucleus (Garibotto et al., 2020).

This study was initiated to investigate [^18^F]nifene binding and nAChR distributions throughout the brain across sex and age cohorts, contributing to the growing understanding of nAChRs and their relevance to addiction and cognitive outcomes. The findings in this study expand upon the results from the previous first-in-human [^18^F]nifene investigations by including nearly four times as many participants, allowing for more robust identification of population differences in [^18^F]nifene binding. The primary area of difference observed, both in age and sex comparison, was in the AN of the thalamus, which is known to have a high density of nAChRs and is functionally associated with cognition, including spatial and episodic memory (Child & Benarroch, 2013). Within the thalamus the AN is a relatively small structure (200 mm^3^) (Möttönen et al., 2015) that is near the resolution of our PET scanners. No partial volume correction was applied to any of our scans in order to limit the variance of our measured PET signal.

A previous study examining [^18^F]nifene binding differences from our group (Mukherjee et al., 2018) reported preliminary age and sex differences in a smaller cohort with four men (age 47.0 ± 17.6) and 4 women (42.8 ± 23.6) with a wide range of ages. Six of these participants were also included in the analysis presented here, although with slightly different ROIs, DVR processing and definition of the CC reference region. While this previous study did not find significant correlation between age and [^18^F]nifene binding in the thalamus or SN, a descriptive increase in DVR in the cerebellum and frontal cortex with age was observed, similar to the findings reported here. Using our method of data processing on the eight images from this initial study, we similarly do not see any significant associations between age and [^18^F]nifene binding in any of the observed regions, including the thalamus. This lack of significance in the thalamus is likely driven by the small sample size and that the two participants with the lowest thalamic DVR in the younger cohort were from this previous study, potentially underrepresenting the expected average [^18^F]nifene binding for the younger cohort in this study’s comparison.

This study focused primarily on demographic differences in [^18^F]nifene binding and α4β2* nAChRs, and thus we did not test or attempt to quantify sex-related hormones in our participants. In a previous study using [^123^I]5-IA-85380 SPECT imaging to measure β_2_* nAChR availability fromCosgrove et al. (2007), female nonsmokers were found to have greater tracer binding throughout the brain, including the frontal lobe, thalamus, and striatum. Additionally, a significant negative correlation was found between progesterone levels and binding in regions, including the cerebellum, although there was not a significant correlation with progesterone levels observed in the thalamus. This other study focused primarily on younger nonsmokers who were age matched for sex-based comparisons compared to ours which included a larger age range, which may have obscured any higher binding of [^18^F]nifene in our model. Further work in addiction studies related to nAChR densities could be improved by obtaining information including birth control status for female participants as well as levels of hormones such as estrogen and progesterone and their correlation with [^18^F]nifene binding for females scanned at different points in the menstrual cycle.

PET imaging and postmortem tissue binding studies have found mixed results regarding the correlation between age and density of nAChRs throughout the brain. In vivo and postmortem investigations have found a reduction in nAChRs with age (Mitsis et al., 2009;Perry et al., 2000;Picciotto & Zoli, 2002), while in others an increase in with age has been shown (Nordberg et al., 1992). A decrease in nAChRs with age in the thalamus was found in this study, the region with the highest [^18^F]nifene binding in the brain. However, the direction of the difference was reversed in the cerebellar GM with higher receptor expression in increasing age. Though unanticipated, we can consider several factors that may be impacting the difference between our older and younger cohorts, including our reference region.

The choice of the CC as a reference region is supported by previous studies using [^18^F]nifene (Betthauser et al., 2017;Lao et al., 2017;Mukherjee et al., 2018) and other nAChR imaging studies (Brody et al., 2006). The use of a white matter reference region when analyzing binding in grey matter regions is admittedly not ideal due to the different nonspecific binding kinetics between white and grey matter with [^18^F]nifene. This can be visualized in the comparison between the nicotine user and an age- and sex-matched study participant (Fig. 4). The nondisplaceable binding in the nicotine user image is higher in grey matter regions, including in regions of interest such as the thalamus and striatum, than in white matter. While the comparison with the nicotine user presented here is inherently limited due to their nicotine challenge not being a part of our study design and only being one participant, similar distributions have been observed with nicotine challenges and other nAChR tracers in human participants such as [^18^F]AZAN (Wong et al., 2013) and [^18^F]2-FA (Brody et al., 2006).

**Fig. 5.**
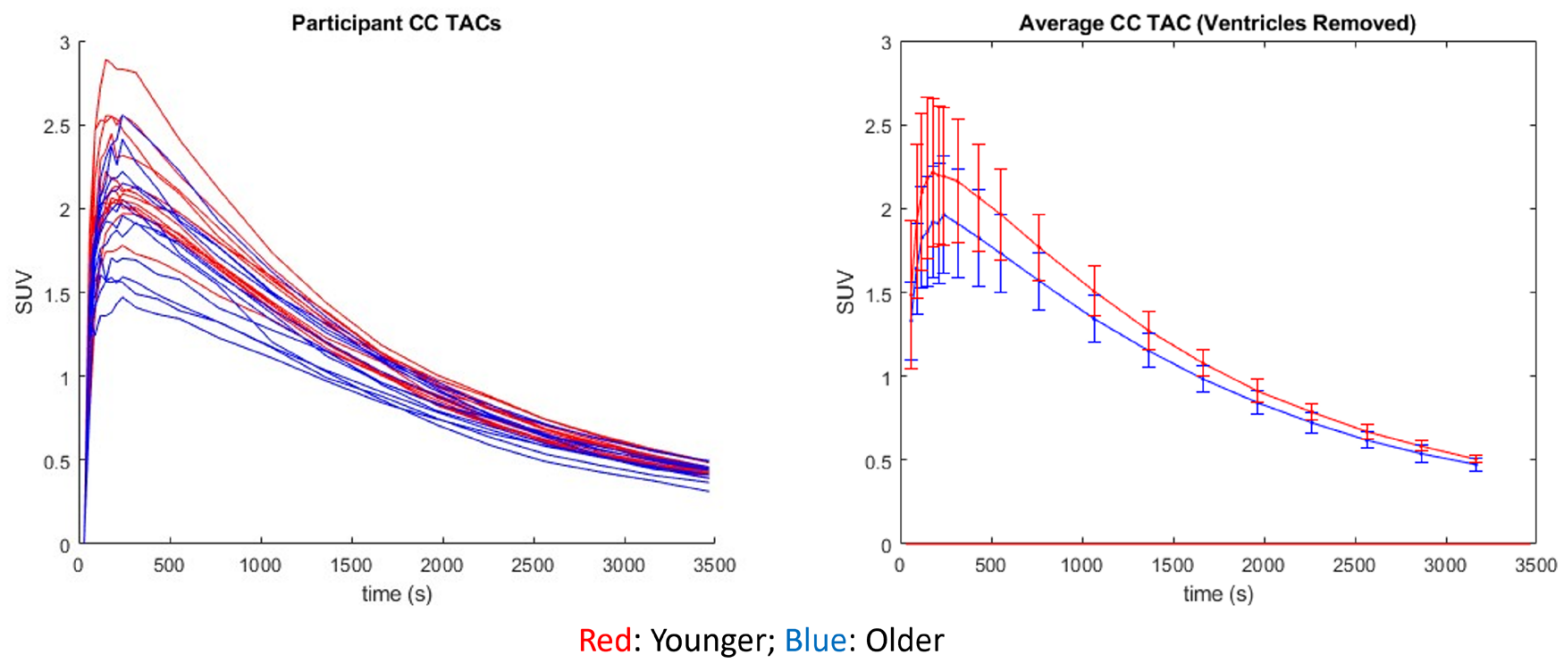
TACs for the individual participants (left) enrolled in our study delineated by color with the older participants in blue and the younger participants in red. The average of these TACs (right) shows the descriptive higher reference region signal in the younger cohort on average.

In studies using (-)-[18F]Flubatine, another α4β2* nAChR PET tracer, researchers found a reduction of tracer distribution volume (*V*_T_) in the CC of over 20% after participants smoked a cigarette (Bhatt et al., 2018). These results indicate the presence of displaceable nAChR tracer binding in the CC, increasing the PET signal measured in this reference region. If there were a reduction of nAChR density with age like the one seen in the thalamus, we would thus expect a higher reference region signal in the younger cohort corresponding to a lower global DVR. Comparing average time activity curves (TACs) between the native space PET scans of the two populations, the younger cohort had a higher average SUV over the course of the scan in all regions analyzed, including the CC reference region (Fig. 5). This is potentially due to a higher brain penetrance of [^18^F]nifene in the younger participants or the imperfect removal of regions of atrophy and ventricle enlargement in the older cohort ROIs; however, when comparing the TACs of the older and younger cohorts, we observed no significant differences between any time points over the course of the scan. Similarly, there was no significant difference observed between the SUV from 40–60 min of the older (2.10 ± 0.21) and younger (2.24 ± 0.14) cohorts in the CC (p = 0.09). The lower average signal in the reference region in the older cohort could explain the generally higher DVR observed with increasing age outside of the thalamus in this study; however, we would not expect this to drive the significant age-related associations with DVR in any of the regions analyzed. Previous work has validated the CC reference region using arterial sampling in rhesus monkeys (Hillmer et al., 2011) and has been used in previous work with [F-18]nifene in humans (Lao et al., 2017) and other nAChR tracers (Brody et al., 2006). No arterial blood sampling was completed for the participants in this study. Thus, it is not possible to characterize potential biases introduced in this DVR outcome measure using an imperfect (WM) reference region.

There are also societal and behavioral differences between age groups related to nicotine exposure that could influence nAChR expression. A previous study using [^18^F]2-FA found α4β2* nAChR density was higher in smokers than in nonsmokers (Mukhin et al., 2008). Although nicotine use was an exclusion criterion for this study (and the one participant providing post-scan affirmation of nicotine use was removed), it is conceivable that environmental exposure to nicotine between these two age groups was likely different throughout their lifetimes. Between 1965 (the first year tracked by the CDC and a year after the birth year of the youngest older participant) and 2018, the percentage of adult smokers decreased from a peak of 42.6% to 13.7% (National Center for Health Statistics, 2023). Smoking in bars and restaurants in Wisconsin was also legal until 2010, when the oldest member of the younger cohort was 16 and the youngest member of the older cohort was 46. Lifetime secondhand nicotine exposure, while not quantified in this study, would likely have been much higher for the older cohort than the younger cohort. However, we acknowledge that this reasoning would not necessarily explain the higher levels of thalamic binding in the younger cohort.

## 5 Conclusions

Significant differences were observed in [^18^F]nifene binding in the thalamus in our cohort population with increasing age. There was a significant decrease in [^18^F]nifene signal with age in the thalamus, most notably in the AN, and a general increase in DVR in the brain regions with lower receptor expression, including the cerebellum GM. These results support the inclusion of age as a consideration in experimental design and data analyses for clinical research investigations of the α4β2* nAChR system.

## Data Availability

PET imaging data from the University of Wisconsin - Madison Alzheimer’s Disease Research Center can be requested using an online application process (https://www.adrc.wisc.edu/apply-resources). Any additional data can be made available upon request.

## References

[b1] Ashburner , J. T. , Friston , K. J. , Kiebel , S. J. , Nichols , T. E. , & Penny , W. D . ( 2011 ). Statistical parametric mapping: The analysis of functional brain images . Elsevier Science . 10.1016/B978-0-12-372560-8.X5000-1

[b2] Beam , C. R. , Kaneshiro , C. , Jang , J. Y. , Reynolds , C. A. , Pedersen , N. L. , & Gatz , M. ( 2018 ). Differences between women and men in incidence rates of dementia and Alzheimer’s disease . J Alzheimers Dis , 64 ( 4 ), 1077 – 1083 . 10.3233/jad-180141 30010124 PMC6226313

[b3] Benowitz , N. L. , & Hatsukami , D. ( 1998 ). Gender differences in the pharmacology of nicotine addiction . Addict Biol , 3 ( 4 ), 383 – 404 . 10.1080/13556219871930 26735114

[b4] Betthauser , T. J. , Hillmer , A. T. , Lao , P. J. , Ehlerding , E. , Mukherjee , J. , Stone , C. K. , & Christian , B. T. ( 2017 ). Human biodistribution and dosimetry of [ ^18^ F]Nifene, an α4β2* nicotinic acetylcholine receptor PET tracer . Nucl Med Biol , 55 , 7 – 11 . 10.1016/j.nucmedbio.2017.08.001 28963927 PMC5709231

[b5] Bhatt , S. , Hillmer , A. T. , Nabulsi , N. , Matuskey , D. , Lim , K. , Lin , S. F. , Esterlis , I. , Carson , R. E. , Huang , Y. , & Cosgrove , K. P. ( 2018 ). Evaluation of (-)-[ ^18^ F]Flubatine-specific binding: Implications for reference region approaches . Synapse , 72 ( 3 ). 10.1002/syn.22016 PMC6547815 29105121

[b6] Brody , A. L. , Mandelkern , M. A. , London , E. D. , Olmstead , R. E. , Farahi , J. , Scheibal , D. , Jou , J. , Allen , V. , Tiongson , E. , Chefer , S. I. , Koren , A. O. , & Mukhin , A. G. ( 2006 ). Cigarette smoking saturates brain alpha 4 beta 2 nicotinic acetylcholine receptors . Arch Gen Psychiatry , 63 ( 8 ), 907 – 915 . 10.1001/archpsyc.63.8.907 16894067 PMC2773659

[b7] Chefer , S. I. , London , E. D. , Koren , A. O. , Pavlova , O. A. , Kurian , V. , Kimes , A. S. , Horti , A. G. , & Mukhin , A. G. ( 2003 ). Graphical analysis of 2-[18F]FA binding to nicotinic acetylcholine receptors in rhesus monkey brain . Synapse , 48 ( 1 ), 25 – 34 . 10.1002/syn.10180 12557269

[b8] Child , N. D. , & Benarroch , E. E. ( 2013 ). Anterior nucleus of the thalamus: Functional organization and clinical implications . Neurology , 81 ( 21 ), 1869 – 1876 . 10.1212/01.wnl.0000436078.95856.56 24142476

[b9] Christian , B. T. , Vandehey , N. T. , Floberg , J. M. , & Mistretta , C. A. ( 2010 ). Dynamic PET denoising with HYPR processing . J Nucl Med , 51 ( 7 ), 1147 – 1154 . 10.2967/jnumed.109.073999 20554743 PMC3250311

[b10] Colloby , S. J. , Perry , E. K. , Pakrasi , S. , Pimlott , S. L. , Wyper , D. J. , McKeith , I. G. , Williams , E. D. , & O’Brien , J. T. ( 2010 ). Nicotinic 123I-5IA-85380 single photon emission computed tomography as a predictor of cognitive progression in Alzheimer’s disease and dementia with Lewy bodies . Am J Geriatr Psychiatry , 18 ( 1 ), 86 – 90 . 10.1097/JGP.0b013e3181b972aa 20094022

[b11] Cosgrove , K. P. , Esterlis , I. , McKee , S. A. , Bois , F. , Seibyl , J. P. , Mazure , C. M. , Krishnan-Sarin , S. , Staley , J. K. , Picciotto , M. R. , & O’Malley , S. S. ( 2012 ). Sex differences in availability of β2*-nicotinic acetylcholine receptors in recently abstinent tobacco smokers . Arch Gen Psychiatry , 69 ( 4 ), 418 – 427 . 10.1001/archgenpsychiatry.2011.1465 22474108 PMC3508698

[b12] Cosgrove , K. P. , Mitsis , E. M. , Bois , F. , Frohlich , E. , Tamagnan , G. D. , Krantzler , E. , Perry , E. , Maciejewski , P. K. , Epperson , C. N. , Allen , S. , O’Malley , S. , Mazure , C. M. , Seibyl , J. P. , van Dyck , C. H. , & Staley , J. K. ( 2007 ). 123I-5-IA-85380 SPECT imaging of nicotinic acetylcholine receptor availability in nonsmokers: Effects of sex and menstrual phase . J Nucl Med , 48 ( 10 ), 1633 – 1640 . 10.2967/jnumed.107.042317 17873128

[b13] Coughlin , J. M. , Slania , S. , Du , Y. , Rosenthal , H. B. , Lesniak , W. G. , Minn , I. , Smith , G. S. , Dannals , R. F. , Kuwabara , H. , Wong , D. F. , Wang , Y. , Horti , A. G. , & Pomper , M. G. ( 2018 ). F-XTRA PET for enhanced imaging of the extrathalamic α4β2 nicotinic acetylcholine receptor . J Nucl Med , 59 ( 10 ), 1603 – 1608 . 10.2967/jnumed.117.205492 29496987 PMC6167533

[b14] Desikan , R. S. , Ségonne , F. , Fischl , B. , Quinn , B. T. , Dickerson , B. C. , Blacker , D. , Buckner , R. L. , Dale , A. M. , Maguire , R. P. , Hyman , B. T. , Albert , M. S. , & Killiany , R. J. ( 2006 ). An automated labeling system for subdividing the human cerebral cortex on MRI scans into gyral based regions of interest . Neuroimage , 31 ( 3 ), 968 – 980 . 10.1016/j.neuroimage.2006.01.021 16530430

[b15] Frazier , J. A. , Chiu , S. , Breeze , J. L. , Makris , N. , Lange , N. , Kennedy , D. N. , Herbert , M. R. , Bent , E. K. , Koneru , V. K. , Dieterich , M. E. , Hodge , S. M. , Rauch , S. L. , Grant , P. E. , Cohen , B. M. , Seidman , L. J. , Caviness , V. S. , & Biederman , J. ( 2005 ). Structural brain magnetic resonance imaging of limbic and thalamic volumes in pediatric bipolar disorder . Am J Psychiatry , 162 ( 7 ), 1256 – 1265 . 10.1176/appi.ajp.162.7.1256 15994707

[b16] Fujita , M. , Al-Tikriti , M. S. , Tamagnan , G. , Zoghbi , S. S. , Bozkurt , A. , Baldwin , R. M. , & Innis , R. B. ( 2003 ). Influence of acetylcholine levels on the binding of a SPECT nicotinic acetylcholine receptor ligand [123I]5-I-A-85380 . Synapse , 48 ( 3 ), 116 – 122 . 10.1002/syn.10194 12645036

[b17] Fujita , M. , Seibyl , J. P. , Vaupel , D. B. , Tamagnan , G. , Early , M. , Zoghbi , S. S. , Baldwin , R. M. , Horti , A. G. , KoreN , A. O. , Mukhin , A. G. , Khan , S. , Bozkurt , A. , Kimes , A. S. , London , E. D. , & Innis , R. B. ( 2002 ). Whole-body biodistribution, radiation absorbed dose, and brain SPET imaging with [123i]5-i-A-85380 in healthy human subjects . Eur J Nucl Med Mol Imaging , 29 ( 2 ), 183 – 190 . 10.1007/s00259-001-0695-z 11926380

[b18] Garibotto , V. , Wissmeyer , M. , Giavri , Z. , Ratib , O. , & Picard , F. ( 2020 ). Nicotinic acetylcholine receptor density in the “higher-order” thalamus projecting to the prefrontal cortex in humans: A PET study . Mol Imaging Biol , 22 ( 2 ), 417 – 424 . 10.1007/s11307-019-01377-8 31140109

[b19] Goldstein , J. M. , Seidman , L. J. , Makris , N. , Ahern , T. , O’Brien , L. M. , Caviness , V. S. , Jr , Kennedy , D. N. , Faraone , S. V. , & Tsuang , M. T. ( 2007 ). Hypothalamic abnormalities in schizophrenia: Sex effects and genetic vulnerability . Biol Psychiatry , 61 ( 8 ), 935 – 945 . 10.1016/j.biopsych.2006.06.027 17046727

[b20] Hillmer , A. T. , Wooten , D. W. , Moirano , J. M. , Slesarev , M. , Barnhart , T. E. , Engle , J. W. , Nickles , R. J. , Murali , D. , Schneider , M. L. , Mukherjee , J. , & Christian , B. T. ( 2011 ). Specific α4β2 nicotinic acetylcholine receptor binding of [F-18]nifene in the rhesus monkey . Synapse , 65 ( 12 ), 1309 – 1318 . 10.1002/syn.20965 21674627 PMC3705633

[b21] Hillmer , A. T. , Wooten , D. W. , Slesarev , M. S. , Ahlers , E. O. , Barnhart , T. E. , Murali , D. , Schneider , M. L. , Mukherjee , J. , & Christian , B. T. ( 2012 ). PET imaging of α4β2* nicotinic acetylcholine receptors: Quantitative analysis of 18F-nifene kinetics in the nonhuman primate . J Nucl Med , 53 ( 9 ), 1471 – 1480 . 10.2967/jnumed.112.103846 22851633 PMC3580212

[b22] Hillmer , A. T. , Wooten , D. W. , Slesarev , M. S. , Ahlers , E. O. , Barnhart , T. E. , Schneider , M. L. , Mukherjee , J. , & Christian , B. T. ( 2013 ). Measuring α4β2* nicotinic acetylcholine receptor density in vivo with [(18)F]nifene PET in the nonhuman primate . J Cereb Blood Flow Metab , 33 ( 11 ), 1806 – 1814 . 10.1038/jcbfm.2013.136 23942367 PMC3824181

[b23] Horti , A. G. , Gao , Y. , Kuwabara , H. , & Dannals , R. F. ( 2010 ). Development of radioligands with optimized imaging properties for quantification of nicotinic acetylcholine receptors by positron emission tomography . Life Sci , 86 ( 15–16 ), 575 – 584 . 10.1016/j.lfs.2009.02.029 19303028 PMC2848883

[b24] Horti , A. G. , Kuwabara , H. , Holt , D. P. , Dannals , R. F. , & Wong , D. F. ( 2013 ). Recent PET radioligands with optimal brain kinetics for imaging nicotinic acetylcholine receptors . J Labelled Comp Radiopharm , 56 ( 3–4 ), 159 – 166 . 10.1002/jlcr.3020 24285321

[b25] Horti , A. G. , Scheffel , U. , Koren , A. O. , Ravert , H. T. , Mathews , W. B. , Musachio , J. L. , Finley , P. A. , London , E. D. , & Dannals , R. F. ( 1998 ). 2-[18F]Fluoro-A-85380, an in vivo tracer for the nicotinic acetylcholine receptors . Nucl Med Biol , 25 ( 7 ), 599 – 603 . 10.1016/s0969-8051(98)00031-6 9804040

[b26] Kaye , J. A. , DeCarli , C. , Luxenberg , J. S. , & Rapoport , S. I. ( 1992 ). The significance of age-related enlargement of the cerebral ventricles in healthy men and women measured by quantitative computed X-ray tomography . J Am Geriatr Soc , 40 ( 3 ), 225 – 231 . 10.1111/j.1532-5415.1992.tb02073.x 1538040

[b27] Kimes , A. S. , Horti , A. G. , London , E. D. , Chefer , S. I. , Contoreggi , C. , Ernst , M. , Friello , P. , Koren , A. O. , Kurian , V. , Matochik , J. A. , Pavlova , O. , Vaupel , D. B. , & Mukhin , A. G. ( 2003 ). 2-[18F]F-A-85380: PET imaging of brain nicotinic acetylcholine receptors and whole body distribution in humans . FASEB J , 17 ( 10 ), 1331 – 1333 . 10.1096/fj.02-0492fje 12759330

[b28] Lao , P. J. , Betthauser , T. J. , Tudorascu , D. L. , Barnhart , T. E. , Hillmer , A. T. , Stone , C. K. , Mukherjee , J. , & Christian , B. T. ( 2017 ). [ ^18^ F]Nifene test-retest reproducibility in first-in-human imaging of α4β2* nicotinic acetylcholine receptors . Synapse , 71 ( 8 ), 10.1002/syn.21981 PMC5541262 28420041

[b29] Lindstrom , J. , Anand , R. , Peng , X. , Gerzanich , V. , Wang , F. , & Li , Y. ( 1995 ). Neuronal nicotinic receptor subtypes . Ann N Y Acad Sci , 757 , 100 – 116 . 10.1111/j.1749-6632.1995.tb17467.x 7611667

[b30] Lodge , M. A. , Leal , J. P. , Rahmim , A. , Sunderland , J. J. , & Frey , E. C. ( 2018 ). Measuring PET spatial resolution using a cylinder phantom positioned at an oblique angle . J Nucl Med , 59 ( 11 ), 1768 – 1775 . 10.2967/jnumed.118.209593 29903932 PMC6225537

[b31] Logan , J. , Fowler , J. S. , Volkow , N. D. , Wang , G. J. , Ding , Y. S. , & Alexoff , D. L. ( 1996 ). Distribution volume ratios without blood sampling from graphical analysis of PET data . J Cereb Blood Flow Metab , 16 ( 5 ), 834 – 840 . 10.1097/00004647-199609000-00008 8784228

[b32] Makris , N. , Goldstein , J. M. , Kennedy , D. , Hodge , S. M. , Caviness , V. S. , Faraone , S. V. , Tsuang , M. T. , & Seidman , L. J. ( 2006 ). Decreased volume of left and total anterior insular lobule in schizophrenia . Schizophr Res , 83 ( 2–3 ), 155 – 171 . 10.1016/j.schres.2005.11.020 16448806

[b33] McKay , B. E. , Placzek , A. N. , & Dani , J. A. ( 2007 ). Regulation of synaptic transmission and plasticity by neuronal nicotinic acetylcholine receptors . Biochem Pharmacol , 74 ( 8 ), 1120 – 1133 . 10.1016/j.bcp.2007.07.001 17689497 PMC2047292

[b34] Meyer , P. M. , Strecker , K. , Kendziorra , K. , Becker , G. , Hesse , S. , Woelpl , D. , Hensel , A. , Patt , M. , Sorger , D. , Wegner , F. , Lobsien , D. , Barthel , H. , Brust , P. , Gertz , H. J. , Sabri , O. , & Schwarz , J. ( 2009 ). Reduced alpha4beta2*-nicotinic acetylcholine receptor binding and its relationship to mild cognitive and depressive symptoms in Parkinson disease . Arch Gen Psychiatry , 66 ( 8 ), 866 – 877 . 10.1001/archgenpsychiatry.2009.106 19652126

[b35] Meyer , P. M. , Tiepolt , S. , Barthel , H. , Hesse , S. , & Sabri , O. ( 2014 ). Radioligand imaging of α4β2* nicotinic acetylcholine receptors in Alzheimer’s disease and Parkinson’s disease . Q J Nucl Med Mol Imaging , 58 ( 4 ), 376 – 386 . https://www.researchgate.net/publication/268230725_Radioligand_imaging_of_a4b2_nicotinic_acetylcholine_receptors_in_Alzheimer’s_disease_and_Parkinson’s_disease 25387119

[b36] Mitsis , E. M. , Cosgrove , K. P. , Staley , J. K. , Bois , F. , Frohlich , E. B. , Tamagnan , G. D. , Estok , K. M. , Seibyl , J. P. , & van Dyck , C. H . ( 2009 ). Age-related decline in nicotinic receptor availability with [(123)I]5-IA-85380 SPECT . Neurobiol Aging , 30 ( 9 ), 1490 – 1497 . 10.1016/j.neurobiolaging.2007.12.008 18242781 PMC3523217

[b37] Möttönen , T. , Katisko , J. , Haapasalo , J. , Tähtinen , T. , Kiekara , T. , Kähärä , V. , Peltola , J. , Öhman , J. , & Lehtimäki , K. ( 2015 ). Defining the anterior nucleus of the thalamus (ANT) as a deep brain stimulation target in refractory epilepsy: Delineation using 3 T MRI and intraoperative microelectrode recording . Neuroimage Clin , 7 , 823 – 829 . 10.1016/j.nicl.2015.03.001 26082891 PMC4459042

[b38] Mukherjee , J. , Lao , P. J. , Betthauser , T. J. , Samra , G. K. , Pan , M. L. , Patel , I. H. , Liang , C. , Metherate , R. , & Christian , B. T. ( 2018 ). Human brain imaging of nicotinic acetylcholine α4β2* receptors using [ ^18^ F]Nifene: Selectivity, functional activity, toxicity, aging effects, gender effects, and extrathalamic pathways . J Comp Neurol , 526 ( 1 ), 80 – 95 . 10.1002/cne.24320 28875553 PMC5788574

[b39] Mukhin , A. G. , Kimes , A. S. , Chefer , S. I. , Matochik , J. A. , Contoreggi , C. S. , Horti , A. G. , Vaupel , D. B. , Pavlova , O. , & Stein , E. A. ( 2008 ). Greater nicotinic acetylcholine receptor density in smokers than in nonsmokers: A PET study with 2-18F-FA-85380 . J Nucl Med , 49 ( 10 ), 1628 – 1635 . 10.2967/jnumed.108.050716 18794265 PMC2766917

[b40] National Center for Health Statistics . ( 2023 ). Health, United States, 2020–2021: Annual Perspective . U.S. Department of Health and Human Services, Centers for Disease Control and Prevention, National Center for Health Statistics . https://www.cdc.gov/nchs/data/hus/hus20-21.pdf

[b41] Nordberg , A. , Alafuzoff , I. , & Winblad , B. ( 1992 ). Nicotinic and muscarinic subtypes in the human brain: Changes with aging and dementia . J Neurosci Res , 31 ( 1 ), 103 – 111 . 10.1002/jnr.490310115 1613816

[b42] O’Brien , J. T. , Colloby , S. J. , Pakrasi , S. , Perry , E. K. , Pimlott , S. L. , Wyper , D. J. , McKeith , I. G. , & Williams , E. D. ( 2008 ). Nicotinic alpha4beta2 receptor binding in dementia with Lewy bodies using 123I-5IA-85380 SPECT demonstrates a link between occipital changes and visual hallucinations . Neuroimage , 40 ( 3 ), 1056 – 1063 . 10.1016/j.neuroimage.2008.01.010 18295510

[b43] O’Dell , L. E. , & Torres , O. V. ( 2014 ). A mechanistic hypothesis of the factors that enhance vulnerability to nicotine use in females . Neuropharmacology , 76 , 566 – 580 . 10.1016/j.neuropharm.2013.04.055 23684991 PMC3812395

[b44] Oishi , K. , Zilles , K. , Amunts , K. , Faria , A. , Jiang , H. , Li , X. , Akhter , K. , Hua , K. , Woods , R. , Toga , A. W. , Pike , G. B. , Rosa-Neto , P. , Evans , A. , Zhang , J. , Huang , H. , Miller , M. I. , van Zijl , P. C. , Mazziotta , J. , & Mori , S. ( 2008 ). Human brain white matter atlas: Identification and assignment of common anatomical structures in superficial white matter . Neuroimage , 43 ( 3 ), 447 – 457 . 10.1016/j.neuroimage.2008.07.009 18692144 PMC2586008

[b45] Okada , H. , Ouchi , Y. , Ogawa , M. , Futatsubashi , M. , Saito , Y. , Yoshikawa , E. , Terada , T. , Oboshi , Y. , Tsukada , H. , Ueki , T. , Watanabe , M. , Yamashita , T. , & Magata , Y. ( 2013 ). Alterations in α4β2 nicotinic receptors in cognitive decline in Alzheimer’s aetiopathology . Brain , 136 ( Pt 10 ), 3004 – 3017 . 10.1093/brain/awt195 23975517

[b46] Perry , E. , Martin-Ruiz , C. , Lee , M. , Griffiths , M. , Johnson , M. , Piggott , M. , Haroutunian , V. , Buxbaum , J. D. , Nãsland , J. , Davis , K. , Gotti , C. , Clementi , F. , Tzartos , S. , Cohen , O. , Soreq , H. , Jaros , E. , Perry , R. , Ballard , C. , McKeith , I. , & Court , J. ( 2000 ). Nicotinic receptor subtypes in human brain ageing, Alzheimer and Lewy body diseases . Eur J Pharmacol , 393 ( 1–3 ), 215 – 222 . 10.1016/s0014-2999(00)00064-9 10771016

[b47] Picciotto , M. R. , & Zoli , M. ( 2002 ). Nicotinic receptors in aging and dementia . J Neurobiol , 53 ( 4 ), 641 – 655 . 10.1002/neu.10102 12436427

[b48] Pichika , R. , Easwaramoorthy , B. , Collins , D. , Christian , B. T. , Shi , B. , Narayanan , T. K. , Potkin , S. G. , & Mukherjee , J. ( 2006 ). Nicotinic alpha4beta2 receptor imaging agents: Part II. Synthesis and biological evaluation of 2-[18F]fluoro-3-[2-((S)-3-pyrrolinyl)methoxy]pyridine (18F-nifene) in rodents and imaging by PET in nonhuman primate . Nucl Med Biol , 33 ( 3 ), 295 – 304 . 10.1016/j.nucmedbio.2005.12.017 16631077

[b49] Pogun , S. , Yararbas , G. , Nesil , T. , & Kanit , L. ( 2017 ). Sex differences in nicotine preference . J Neurosci Res , 95 ( 1–2 ), 148 – 162 . 10.1002/jnr.23858 27870459

[b50] Sabri , O. , Becker , G. A. , Meyer , P. M. , Hesse , S. , Wilke , S. , Graef , S. , Patt , M. , Luthardt , J. , Wagenknecht , G. , Hoepping , A. , Smits , R. , Franke , A. , Sattler , B. , Habermann , B. , Neuhaus , P. , Fischer , S. , Tiepolt , S. , Deuther-Conrad , W. , Barthel , H. , … Brust , P . ( 2015 ). First-in-human PET quantification study of cerebral α4β2* nicotinic acetylcholine receptors using the novel specific radioligand (-)-[(18)F]Flubatine . Neuroimage , 118 , 199 – 208 . 10.1016/j.neuroimage.2015.05.065 26037057

[b51] Saranathan , M. , Iglehart , C. , Monti , M. , Tourdias , T. , & Rutt , B. ( 2021 ). In vivo high-resolution structural MRI-based atlas of human thalamic nuclei . Sci Data , 8 ( 1 ), 275 . 10.1038/s41597-021-01062-y 34711852 PMC8553748

[b52] Sultzer , D. L. , Lim , A. C. , Gordon , H. L. , Yarns , B. C. , & Melrose , R. J. ( 2022 ). Cholinergic receptor binding in unimpaired older adults, mild cognitive impairment, and Alzheimer’s disease dementia . Alzheimers Res Ther , 14 ( 1 ), 25 . 10.1186/s13195-021-00954-w 35130968 PMC8819935

[b53] Terry , A. V. , & Callahan , P. M. ( 2019 ). Nicotinic acetylcholine receptor ligands, cognitive function, and preclinical approaches to drug discovery . Nicotine Tob Res , 21 ( 3 ), 383 – 394 . 10.1093/ntr/nty166 30137518 PMC6379039

[b54] U.S. Department of Health and Human Services . ( 2014 ). The health consequences of smoking—50 years of progress: A report of the surgeon general. U.S. Department of Health and Human Services, Centers for Disease Control and Prevention, National Center for Chronic Disease Prevention and Health Promotion, Office on Smoking and Health . https://www.hhs.gov/sites/default/files/consequences-smoking-exec-summary.pdf

[b55] Verplaetse , T. L. , Morris , E. D. , McKee , S. A. , & Cosgrove , K. P. ( 2018 ). Sex differences in the nicotinic acetylcholine and dopamine receptor systems underlying tobacco smoking addiction . Curr Opin Behav Sci , 23 , 196 – 202 . 10.1016/j.cobeha.2018.04.004 31341936 PMC6656369

[b56] Wong , D. F. , Kuwabara , H. , Kim , J. , Brasic , J. R. , Chamroonrat , W. , Gao , Y. , Valentine , H. , Willis , W. , Mathur , A. , McCaul , M. E. , Wand , G. , Gean , E. G. , Dannals , R. F. , & Horti , A. G. ( 2013 ). PET imaging of high-affinity α4β2 nicotinic acetylcholine receptors in humans with 18F-AZAN, a radioligand with optimal brain kinetics . J Nucl Med , 54 ( 8 ), 1308 – 1314 . 10.2967/jnumed.112.108001 23801676

[b57] Woolrich , M. W. , Jbabdi , S. , Patenaude , B. , Chappell , M. , Makni , S. , Behrens , T. , Beckmann , C. , Jenkinson , M. , & Smith , S. M. ( 2009 ). Bayesian analysis of neuroimaging data in FSL . Neuroimage , 45 ( 1 Suppl ), S173 – 186 . 10.1016/j.neuroimage.2008.10.055 19059349

[b58] Zammit , M. , Kao , C. M. , Zhang , H. J. , Tsai , H. M. , Holderman , N. , Mitchell , S. , Tanios , E. , Bhuiyan , M. , Freifelder , R. , Kucharski , A. , Green , W. N. , Mukherjee , J. , & Chen , C. T. ( 2023 ). Evaluation of an image-derived input function for kinetic modeling of nicotinic acetylcholine receptor-binding PET ligands in mice . Int J Mol Sci , 24 ( 21 ), 15510 . 10.3390/ijms242115510 37958495 PMC10650787

[b59] Zhang , H. J. , Zammit , M. , Kao , C. M. , Govind , A. P. , Mitchell , S. , Holderman , N. , Bhuiyan , M. , Freifelder , R. , Kucharski , A. , Zhuang , X. , Mukherjee , J. , Chen , C. T. , & Green , W. N. ( 2023 ). Trapping of nicotinic acetylcholine receptor ligands assayed by in vitro cellular studies and in vivo PET imaging . J Neurosci , 43 ( 1 ), 2 – 13 . 10.1523/JNEUROSCI.2484-21.2022 36028313 PMC9838697

